# Core-Shell Dual-Gate Nanowire Charge-Trap Memory for Synaptic Operations for Neuromorphic Applications

**DOI:** 10.3390/nano11071773

**Published:** 2021-07-07

**Authors:** Md. Hasan Raza Ansari, Udaya Mohanan Kannan, Seongjae Cho

**Affiliations:** Graduate School of IT Convergence Engineering, Gachon University, Seongnam 13120, Korea; hasanrazaadnan@gmail.com (M.H.R.A.); kannan.um@gmail.com (U.M.K.)

**Keywords:** short-term potentiation (STP), long-term potentiation (LTP), charge-trap synaptic transistor, band-to-band tunneling, pattern recognition, neural network, neuromorphic system

## Abstract

This work showcases the physical insights of a core-shell dual-gate (CSDG) nanowire transistor as an artificial synaptic device with short/long-term potentiation and long-term depression (LTD) operation. Short-term potentiation (STP) is a temporary potentiation of a neural network, and it can be transformed into long-term potentiation (LTP) through repetitive stimulus. In this work, floating body effects and charge trapping are utilized to show the transition from STP to LTP while de-trapping the holes from the nitride layer shows the LTD operation. Furthermore, linearity and symmetry in conductance are achieved through optimal device design and biases. In a system-level simulation, with CSDG nanowire transistor a recognition accuracy of up to 92.28% is obtained in the Modified National Institute of Standards and Technology (MNIST) pattern recognition task. Complementary metal-oxide-semiconductor (CMOS) compatibility and high recognition accuracy makes the CSDG nanowire transistor a promising candidate for the implementation of neuromorphic hardware.

## 1. Introduction

Modern day computer architectures suffer from the Von Neumann bottleneck where the separation of memory and processing units impose a fundamental limit on the maximum achievable processing speeds. In addition, the high levels of energy consumption in the conventional computing architecture are a major drawback especially for data intensive applications like big data analytics, machine learning etc. The human brain on the other hand has a highly energy efficient design where the storage and processing are carried out locally using a hugely parallel network of neurons and synapses [1,2]. Neuromorphic systems are gaining research attention due to their potential to design computer chips that can mimic the human brain in merging memory and processing [1,2]. The brain functions (observation, reorganization, learning, and memorization) are performed by neurons (computing elements) and synapses (memory elements) [1,2]. In the neuromorphic system, an artificial synaptic device plays a key role in linking the artificial neurons and modulating the connection strength (synaptic weight) between neurons [3,4,5,6,7,8,9,10,11,12,13,14,15]. In order to realize brain-like computing, different types of artificial synaptic devices have been proposed for artificial intelligence applications [3,4,5,6,7,8,9,10,11,12,13,14,15,16,17,18,19,20,21,22]. The major applications for these artificial synaptic transistors are neuromorphic in-memory computing chip, artificial sensory perception, humanoid robotics, memorize, and recognize massive and unstructured data through parallel and power-efficient ways [3,4,5,6,7,8,9,10,11,12,13,14,15,16,17,18,19,20,21,22]. Charge tapping/de-trapping based artificial synapse are favorable for in-memory computing applications due to their stable analogue conductance state and nonvolatile characteristic [12].

Among these electronic artificial synapses, two terminal non-volatile memory devices such as resistive random access memory (RRAM) and phase change memory (PCM) are strong candidates due to their small form factor [5,6,21,22]. However, due to variability and reliability issues in these devices, the recognition rate undergoes fast degradation. These issues can be resolved with synaptic devices based on complementary metal-oxide-semiconductor (CMOS) field-effect transistors (FET) [15,16,17,18,19,20]. These FET-based electronic synaptic devices operate with a charge trap layer, which is an attractive candidate with many advantages, (i) low synaptic current; (ii) good reliability; (iii) high integration density; (iv) a large conductance window; and (v) process compatibility with CMOS [15,16,17,18,19,20]. These FET-type devices show the feasibility of artificial synapse but may have difficulties in scaling due to short channel effect and band-to-band tunneling in nanoscale regime. These effects degrade the performance of a synaptic transistor and reduces state retention. These issues can be resolved with multigate transistors [23]. Among these transistors, gate all around (GAA) transistor shows the better performance due better controllability over the gate [23,24]. Recently, a novel GAA transistor utilizing a nanotube with a core gate has been proposed to improve the gate controllability and enhance the device performances with same effective silicon film thickness [24,25,26,27,28] and also deal with better scaling over the nanowires [24,25].

Therefore, in this work, we emulate biological synaptic properties such as short-term potentiation (STP), long-term potentiation (LTP), and long-term depression (LTD) in an artificial synaptic device with a novel core-shell gate all around transistor. In neuromorphic systems, STP plays a key role in learning mechanism of the human brain [1,2]. STP is observed due to floating body effect (non-volatile characteristic) and LTP and LTD is observed due to trapping and detrapping of the holes from the nitride layer (volatile characteristic). In order to evaluate the inference capabilities of the proposed synaptic device, the weights (conductance values) extracted from the LTP/LTD characteristics of the device are utilized for pattern recognition using a simulated artificial neural network. The designed neural network recorded a high degree of recognition accuracy of 92.28% for the synaptic device.

## 2. Device Design Strategies and Models Calibration

**Figure 1:**
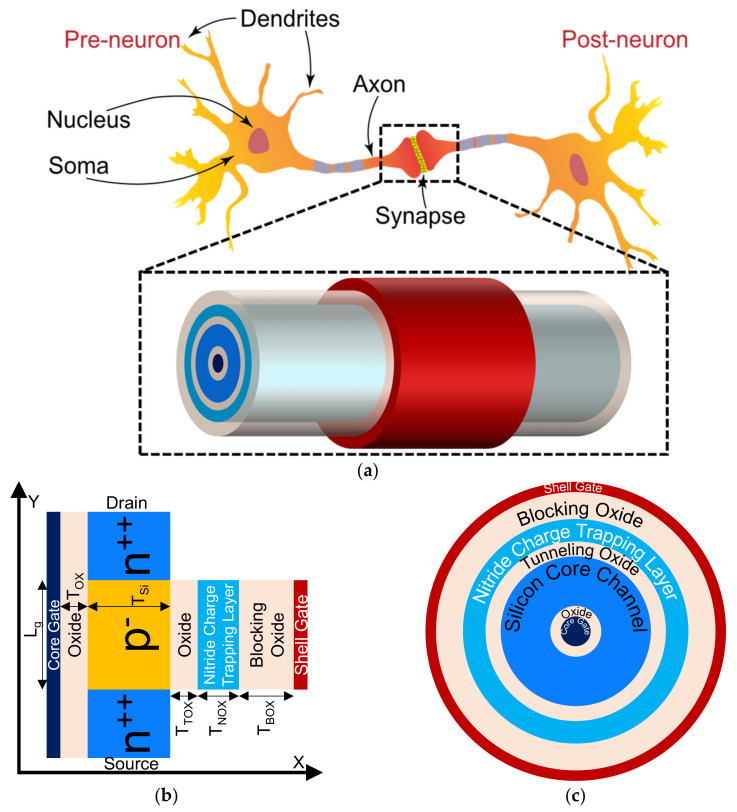
(**a**) Schematic representation of biological synapse and 3D illustration of core-shell dual-gate (CSDG) nanowire transistor. Schematic representation in (**b**) 2D and (**c**) top view of CSDG nanowire transistor for artificial synapse device.

**Figure 2:**
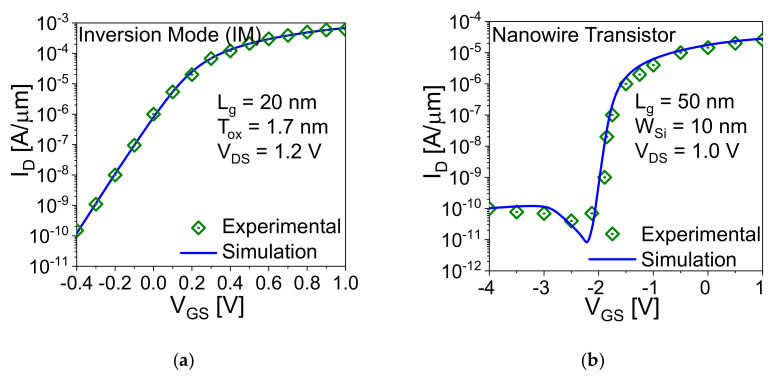
Comparison of simulated transfer characteristic with experimental (**a**) double-gate inversion mode [29] and (**b**) nanowire transistor [30].

Figure 1a–c show the schematic representation of the core-shell dual-gate (CSDG) nanowire transistor in 3D and biological synapse, 2D and top view, respectively. The simulated device dimensions and parameters are optimized and illustrated in Table 1. The device consists of two gates (core (inner) and shell (outer) gate) in gate all around or surrounded gate manner, which helps to accumulate the carriers from the silicon film and makes the deeper potential well for charge storage [24,25,26,27,28]. The dual gate in the device increases the gate controllability and shows better scalability [24,25,26,27,28]. In this work, core gate with thinner oxide (SiO_2_ of 2 nm), which is responsible for the floating body effect for STP while thicker oxide (SiO_2_ of 6 nm/nitride of 4 nm/SiO_2_ of 2 nm (O/N/O)) is utilized for charge trapping to show LTP and LTD operations [17]. The device is simulated with the Silvaco ATLAS (Santa Clara, CA, USA) simulation tool with calibrated models. The physical simulation models are calibrated with experimental transfer characteristics of gate all around and double gate inversion mode transistor as shown in Figure 2a,b, respectively.

Synaptic transistor operations (STP, LTP, and LTD) are based on charge generation, recombination, trapping, and detrapping of charge from the device [15,16,17]. In order to generate the charge in the device, non-local band-to-band-tunneling (BTBT) and impact ionization models are incorporated while for trapping and detrapping the charge from the nitride layer, a macro model (DYNASONOS) is embedded in the simulation, along with hot carrier injection, Fowler–Nordheim (F–N) tunneling, and Poole–Frenkel emission models have also been activated. Other physical models are also incorporated such as the Fermi–Dirac statistics model, concentration-dependent, Shockley–Read–Hall generation, and recombination model, Auger recombination model, density gradient quantum effect, bandgap narrowing model, concentration and temperature-dependent carrier lifetime model, Lombardi’s mobility model.

## 3. Results and Discussion

**Figure 3:**
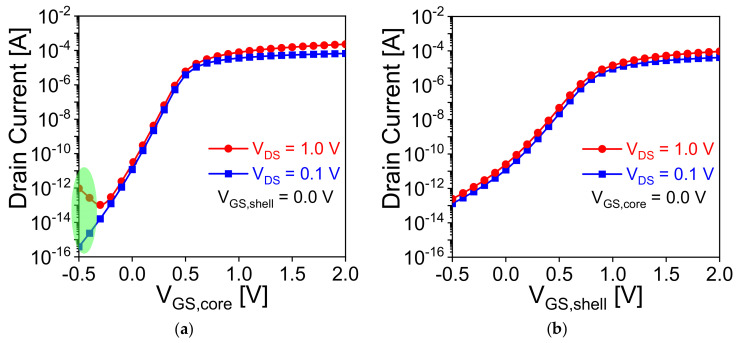
Dual-gate operations of the synaptic memory device. Transfer characteristics at *V*_DS_ = 0.1 V and 1 V. (**a**) *I*_D_-*V*_GS,core_ curves at *V*_GS,shell_ = 0 V. (**b**) *I*_D_-*V*_GS,shell_ curves at *V*_GS,core_ = 0 V. *L*_g_ = 100 nm and *T*_Si_ = 20 nm.

Thanks to core-shell dual gate nanowire transistor, which creates a deeper potential well for charge storage and enhances the retention characteristic of a capacitorless dynamic random access memory (1T DRAM) cell [31]. In this work, the biasing and timing schemes are optimized to achieve STP, LTP, and LTD in the device and mimic the core-shell dual gate transistor as an electronic synapse. The working principle of the device as synapse is based on floating body effect and charge trapping and de-trapping from the nitride layer [15,16,17,32,33]. These operations are based on band-to-band-tunneling, impact ionization, hot carrier injection, and F–N tunneling in the device [15,16,17,19,32,33]. In this work, program operation is based on the BTBT mechanism due to its low power consumption and better reliability issue. In order to achieve the transition from STP to LTP at ≥ 10th pulse, core-gate voltage is optimized with fixed drain voltage of 1.4 V and shell gate voltage of −1.0 V. Achieving STP in the device not only shows the capability for both STP and LTP, but also consumes lower voltage for LTP operation [19]. The reason for the optimization of the core-gate voltage is that band-to-band-tunneling in the device takes place near the channel and drain due to thinner oxide for core-gate. Figure 3a,b show the transfer characteristics of the device with independent gate operation, respectively. It is clearly observed from Figure 3a (drain current-core-gate voltage) that at lower gate voltage, drain current is increasing due to tunnelling between channel and drain compared to Figure 3b (drain current-shell-gate voltage).

**Figure 4:**
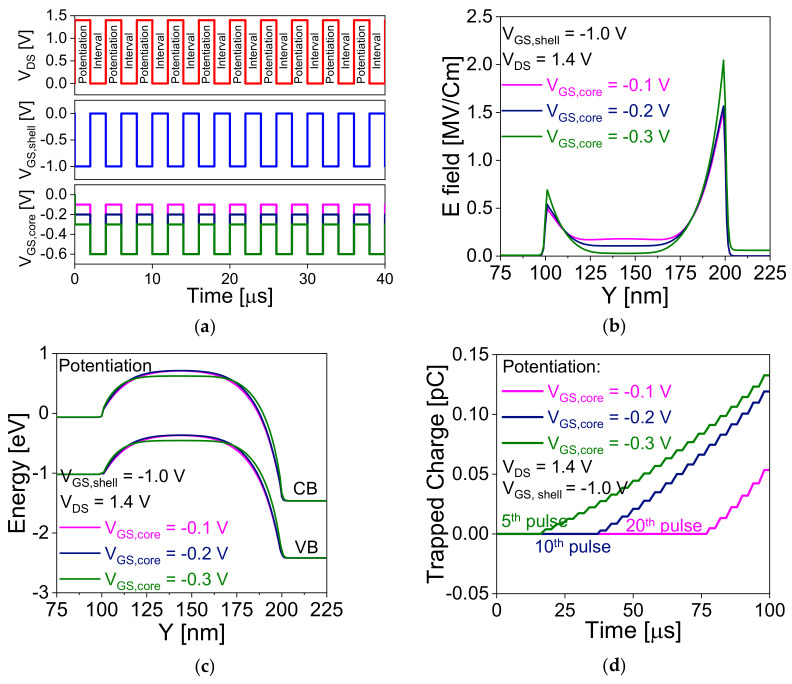
Potentiation operation. (**a**) voltage waveform during potentiation operation. Variation of (**b**) electric field (E filed) and (**c**) energy band diagram with core-gate voltage along the y-direction. (**d**) Variation of trapped charge for different *V*_GS,core_. E field and energy band diagram are extracted 1 nm below of the core gate oxide.

**Figure 5:**
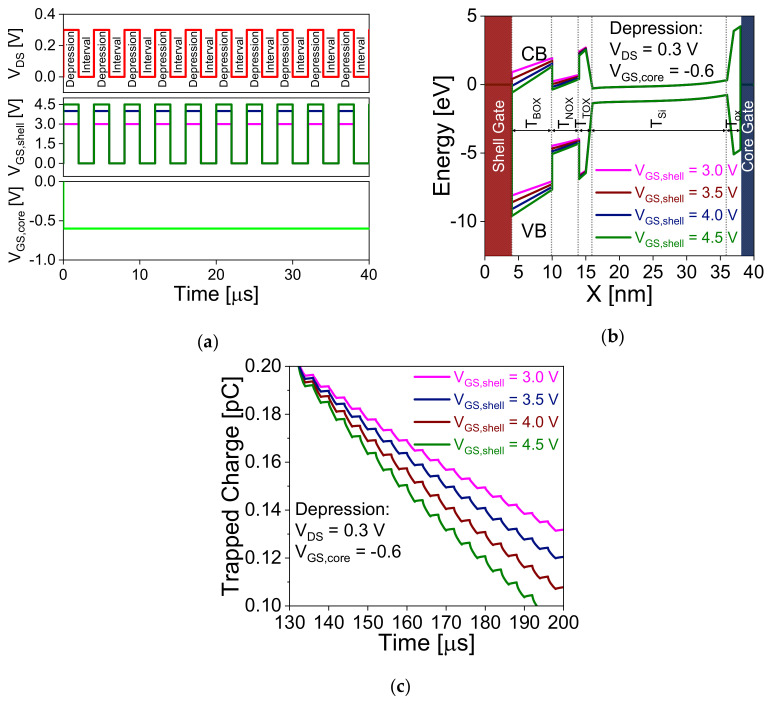
Depression. (**a**) voltage waveform during depression operation. Variation of (**b**) energy band diagram with core-gate voltage along the *x*-direction. (**c**) variation of trapped charge for different *V*_GS,shell_.

Figure 4a shows the voltage waveform during potentiation operation to find the optimized bias with fixed drain and shell gate voltage. A repetitive pulse with pulse and interval width of 2 μs are applied to mimic the device as synapse and shows the transformation from STP to LTP through trapping the charge in the nitride layer. To achieve efficient neuromorphic computational functions, 35 consecutive pulses are applied to stimulate potentiation and, then 35 pulses for depression. Figure 4b,c show the variation of electric field (E field) and energy band diagram, respectively, with different core-gate voltages (−0.1 V, −0.2 V, and −0.3 V). The E field and energy band diagram extracted 1 nm below core gate oxide. CB and VB indicate the conduction and valence band energies. Figure 4b,c reveal that increase in core-gate voltage (in negative magnitude) increases the E field and reduces the tunneling width between the core-gate and drain junctions, which helps to enhance the tunneling in the device and generates more electron hole pairs. The generated holes are stored in the lower potential region, and furthermore, these stored holes trigger the impact ionization in the device and start trapping the holes in the nitride layer. In Figure 4c, the barrier between source and channel is lower for *V*_GS,core_ of −0.3 V compared to lower gate bias. This confirms that the stored holes contribute positive potential and trigger impact ionization in the device and achieves transition at lower pulse as shown in Figure 4d. Figure 4d shows the variation of trapped charge in the nitride layer during potentiation pulse. Similarly, in the case of LTD, shell gate voltage plays a crucial role to de-trap the holes from the nitride layer. Figure 5a shows the voltage waveform during the depression operation to de-trap the charge. Figure 5b shows the energy band diagram of the device during depression operation with different shell gate voltage. Increase in shell gate voltage increases the F–N tunneling probability in the device and starts de-trapping the trapped charges from the nitride layer as shown in Figure 5c.

**Figure 6:**
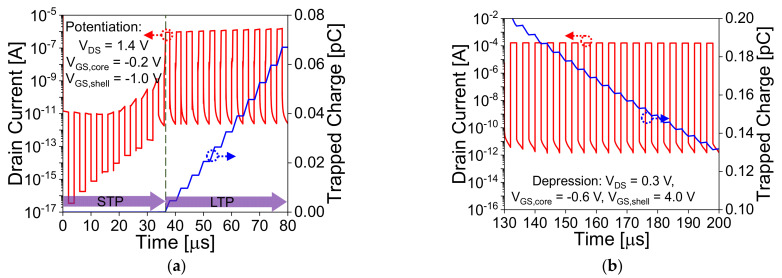
Transient analysis and trapped charges in the nitride layer during (**a**) potentiation and (**b**) depression. A sharp transition in current and trapped charges in the transient analysis of potentiation shows the transformation from short-term potentiation (STP) to long-term potentiation (LTP).

**Figure 7:**
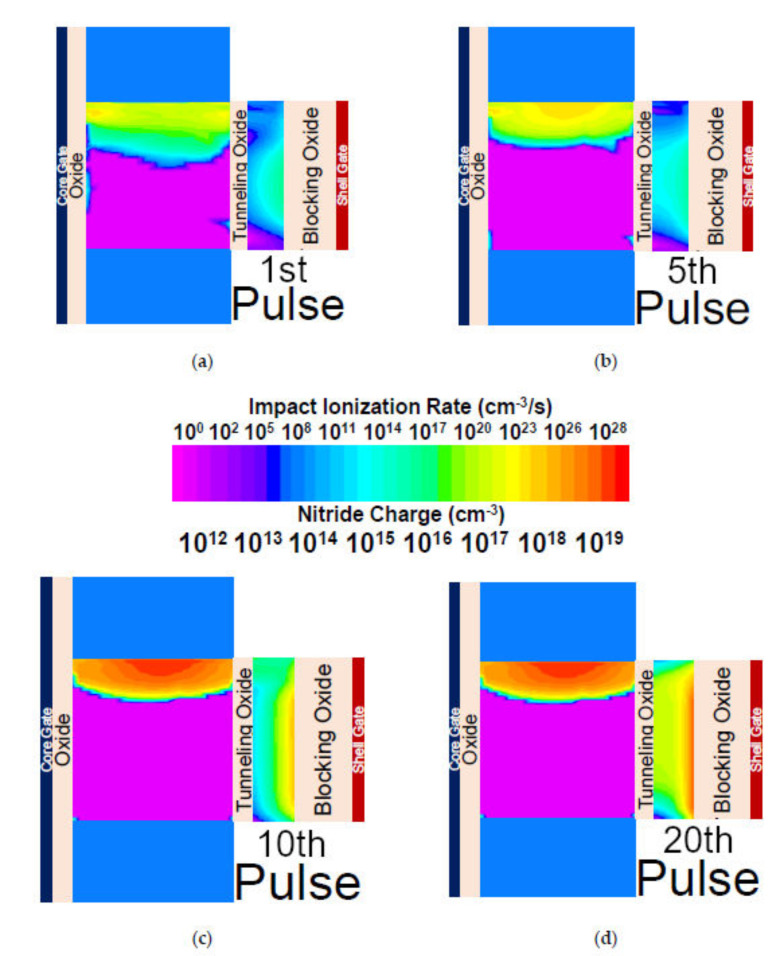
Contour plots of Impact Ionization and charge trapped in the nitride layer during potentiation at (**a**) 1st, (**b**) 5th, (**c**) 10th, and (**d**) 20th pulse.

Figure 6a,b show the transient analysis and trapped charge in the nitride layer of the device during potentiation and depression operation, respectively. During potentiation, a repetitive pulse start trapping the holes in the nitride layer due to hot hole injection and F-N tunnelling at lower bias, which shows the transformation from STP to LTP at the 10th pulse. At the 10th pulse, a sharp transition is observed in the drain current and nitride layer, which confirms that the device is in LTP state. Figure 7a–d show the contour plots of impact ionization rate in the device and charge trapped in the nitride layer during potentiation operation at different pulses (1st pulse, 5th pulse, 10th pulse, and 20th pulse). Initially (1st pulse) for STP, the BTBT mechanism is utilized to generate the holes in the device by applying drain voltage (*V*_DS_) = 1.4 V, core gate voltage (*V*_GS,core_) = −0.2, and shell gate voltage (*V*_GS,shell_) = −1.0 V. The generated holes are accumulated at a lower potential region and electrons start drifting towards the drain side. Further, on increasing the number of repetitive pulses, electrons obtain sufficient energy to trigger the impact ionization in the device and generates more number of electrons-holes pairs in the device. At the 10th pulse, the generated holes get sufficient energy to get trapped in the nitride layer due to F–N tunneling even at lower bias. At the 20th pulse, it can be observed that impact ionization rate is constant while trapped charge in the nitride layer is increasing with increase in pulse. This confirms that after the 10th pulse, generated holes with the energy at the Fermi-Dirac distribution tail have higher probabilities of injection into nitride layer due to hot hole injection. De-trapping the holes from the nitride layer is performed through F–N tunnelling. LTD operation is performed by applying a lower drain bias, *V*_DS_ = 0.3 V, *V*_GS,core_ = −0.6, and higher *V*_GS,shell_ = 4.0 V.

**Figure 8:**
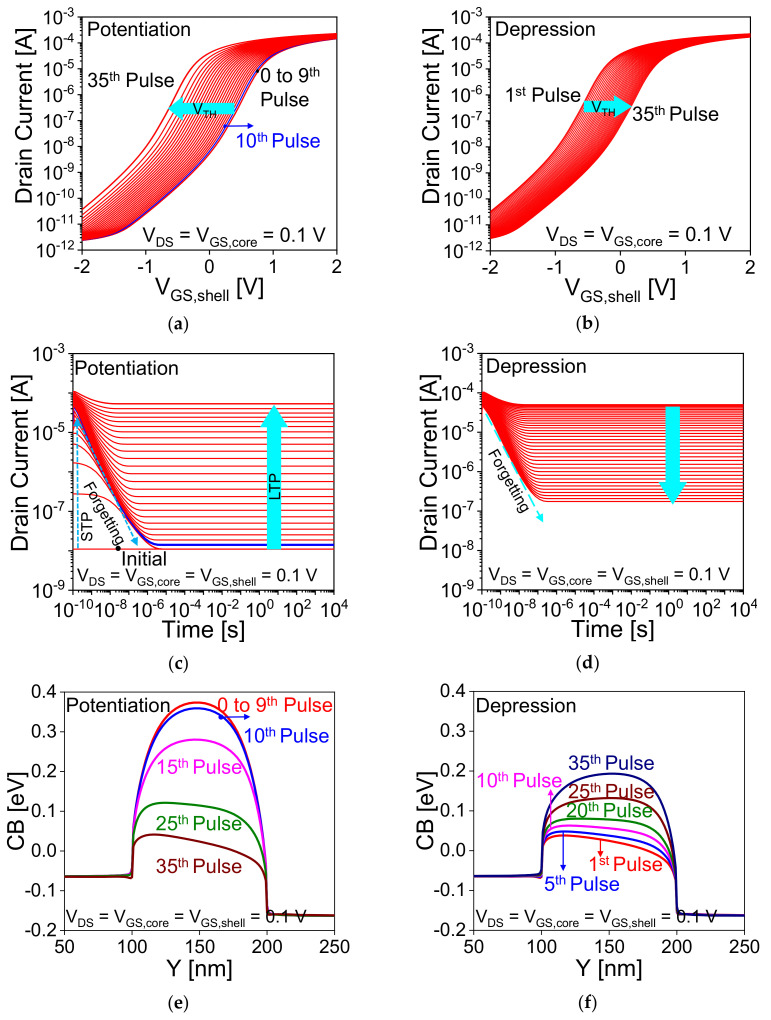
Transfer characteristics of the device during inference (read) operation for (**a**) potentiation (**b**) depression at different pulses. Transient analysis during read operation for (**c**) potentiation (**d**) depression at different pulses. Variation in conduction band (CB) during inference operation for different (**e**) potentiation and (**f**) depression pulses.

The transformation from STP to LTP can also be observed from the transfer characteristics (Figure 8a,b) and transient analysis (Figure 8c,d) of the device during inference (read) operation. Inference in the biological brain is analogous to the read operation in an artificial synaptic transistor. To avoid the disturbance and for non-destructive read a lower bias is applied. Figure 8a,b show the drain current—shell gate voltage curve at *V*_DS_ = *V*_GS,core_ = 0.1 V for different pulses of potentiation and depression, respectively. In the case of potentiation operation, as shown in Figure 8a the transfer characteristics of the device are unchanged during STP states (from 0 to 9th pulse) while from the 10th pulse (LTP), threshold voltage (*V*_TH_) is decreasing with increase in pulse due to increase in trapped charge in the nitride layer (Figure 4d and Figure 6a).

The trapped charges in the nitride layer lower the *V*_TH_ due to the increase in channel potential (lower the barrier for electron), and hence increases the drain current (higher weight) with increase in pulse. Figure 8e,f show the CB energy diagram during inference operation for different potentiation and depression pulses, respectively. Conduction band energies are extracted at below 1 nm of oxide/nitride/oxide (O/N/O) at *V*_DS_ = *V*_GS,core_ = *V*_GS,shell_ = 0.1 V. The same is sensed from Figure 8c for STP that drain current increases for a short time and start decreasing (forgetting) due to recombination of carriers during interval in the device, and thus approaches to the initial level (no pulse (device is at equilibrium condition)). For LTP (from the 10th pulse), current is higher than the STP current and retained up to 10^4^ s due to trapped holes in the nitride layer, which helps to increase the channel potential during inference operation. These results (Figure 8a,c) confirm that this CSDG nanowire transistor is capable of both STP and LTP functions. In the case of LTD, the reverse process is observed, increase in repetitive pulse reduces the channel potential (increases the barrier as shown in Figure 8f) of the device due to de-trapping the holes from the nitride layer (Figure 5c and Figure 6b), and hence increases the threshold voltage (lower weight) of the device as shown in Figure 8c. The same is illustrated in Figure 8d, increase in pulse reduces the current due to recombination of the carriers in the device, and thus current approaches to the initial state with increasing pulse.

**Figure 9:**
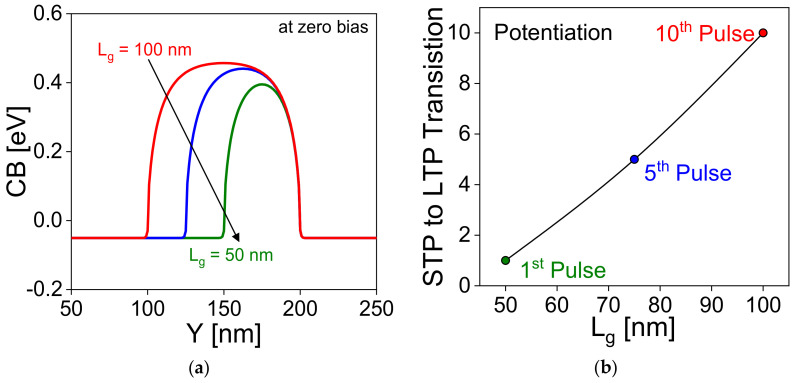
Downscaling of gate length. (**a**) Variation of CB energy. (**b**) number of pulses required for the transformation from STP to LTP. CB and VB indicate conduction and valence band, respectively.

**Figure 10:**
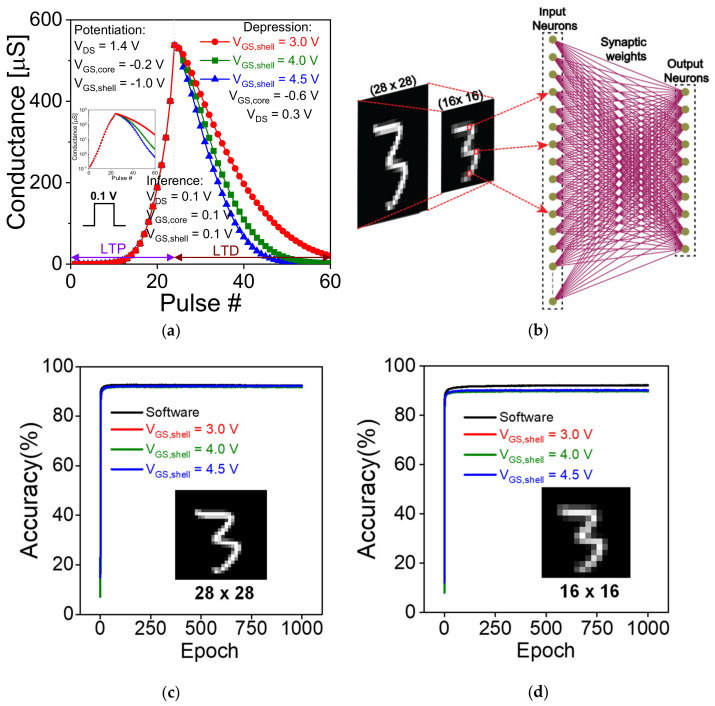
(**a**)Variation in conductance value for Long term potentiation (LTP) and Long-term depression (LTD) characteristics of the CSDG device (**b**) schematic of the single layer neural network with the CSDG transistor as the synapse for Modified National Institute of Standards and Technology (MNIST) digit recognition. Digit recognition accuracy (%) as a function of the number of training epochs at three distinct depression voltages of the synaptic device for (**c**) 28 × 28 and (**d**) 16 × 16 MNIST dataset. Inset of (**c**,**d**) shows the MNIST image of digit “3” in 28 × 28 and 16 × 16 resolutions, respectively.

Figure 9a shows the conduction band (CB) diagram at zero bias condition for different gate length (100 nm, 75 nm, and 50 nm). Reduction in gate length reduces the storage area for floating based memory, which reduces the retention time [34,35,36,37,38,39]. The operation of this synaptic transistor is based on the floating body effect, and charge trapping/de-trapping from the nitride layer. Thus, reduction in gate length reduces the minimum required potentiation pulses by which STP-to-LTP transit occurs as a function of gate length. Downscaling of gate length increases the electric field between the channel and source/drain junctions, which increases the tunneling in the device, and thus, minimum number of pulses are required for the STP-to-LTP transition decreases as shown in Figure 9b. As we scale down the device dimensions, the potentiation behaviour of the device remains the same except for the reduction in pulse number required for STP to LTP transition. Figure 10a shows the variation of conductance (weight) value of LTP and LTD operations with different pulses, respectively. In inset of Figure 10a shows the conductance value in logarithmic scale. From Figure 6b, it is evident that the conductance value for LTP operation is relatively linear compared to the LTD state because the charge trapped in the nitride layer is increasing logarithmically with an increase in the number of pulses (Figure 4d and Figure 6a). In the case of LTD operation, conductance value is estimated with different shell gate voltage (*V*_GS,shell_ of 3.0, 4.0, and 4.5 V). This shows that for shell gate voltage of 4.5 V, more number of holes are de-trapped from the nitride layer, and thus reduces the conductance value abruptly. For shell gate voltage of 4.0 V, the conductance is linear compared to 3.0 and 4.5 V because the amount holes de-trapped rate from the nitride layer is lower. Although an increase in *V*_GS,shell_ > 4.5 V (increasing the F–N tunneling probability), increases the memory window during depression but degrades the conductance value abruptly. In the same way, the LTP weight can also be modulated, and it is more prominent by core gate voltage (*V*_GS,core_) because the tunneling is govern by (*V*_GS,core_). Thus, to obtain the linear conductance value, voltages during operations (potentiation, depression, and inference) need to be optimized. The linearity in the conductance of the LTP and LTD curves affects the inference accuracy of the neural network because it is related to the degree of the synaptic weight change.

Finally, to investigate the learning and inference capabilities of the proposed synaptic device for hardware-based neural networks (HNN), we have simulated a single layer neural network (NN) [40] consisting of one input layer and one output layer as shown in Figure 10b. The synaptic weights (conductance values) obtained from the Potentiation/Depression data in Figure 10a was used for off-chip training of the NN. The designed NN was used to classify image data from a Modified National Institute of Standards and Technology (MNIST) dataset which consists of 60,000 training images and 10,000 test images of handwritten digits from “0” to “9”. All the images are in grayscale format with a resolution of 28 × 28 pixels. The normalized pixel intensities in the interval [0,1] are linearized to form a column matrix with 784 elements which is then fed to the input of the NN. The input values ($x_{i}$) undergo vector matrix multiplication [27] with the corresponding weight values ($w_{ij}$) and is summed up to form $\sum_{i,j}w_{ij}x_{i}$ at each of the output neurons. *i* and *j* denote the number of input nodes and output nodes, respectively. The output at each of these neurons is then transformed using a rectified linear unit (ReLU) activation function. The output neuron with the highest probability for a particular input image is considered as the corresponding prediction from the NN. In the present work, we have also trained the NN using a 16 × 16 downscaled version of the MNIST dataset, so that the effective number of input nodes is reduced to 256. Figure 10c,d shows the variation of accuracy of digit recognition with the number of epochs for the 28 × 28 MNIST dataset using randomly initialized weight distribution (software-based) and device weights extracted for 3 distinct values of depression voltages (V_GS__,shell_) i.e., 3.0 V, 4.0 V and 4.5 V. The final accuracy of digit recognition for the weight update from devices with *V*_GS__,shell_ = 3.0 V, 4.0 V and 4.5 V after 1000 epochs was 91.88%, 91.91% and 92.28% respectively which is very close to the ideal software based NN accuracy of 92.36%. This high accuracy in image recognition reveals that the proposed synaptic device is highly suited for neuromorphic inference applications. Similarly, 16 × 16 MNIST images were used for the devices with *V*_GS__,shell_ = 3.0 V, 4.0 V and 4.5 V yielding an accuracy of 89.94%, 89.65% and 90.17% respectively. In comparison to the software-based recognition accuracy of 92.25%, there is only a marginal drop in accuracy for these devices, which indicates the high reproducibility of our synaptic devices for inference applications requiring reduced input data.

## 4. Conclusions

In this work, we have simulated a novel core-shell dual gate nanowire transistor as an artificial synaptic transistor with calibrated simulation models. The dual gate helps to achieve a deeper potential well for charge retention of the device. The analysis highlights that the combination of floating body effect and charge trapped in the nitride achieves short-term potentiation and long-term potentiation and depression. The results of the CSDG nanowire indicate the following.

1. Transformation from STP to LTP occurs at the 10th pulse and it can be modulated through core gate voltage (*V*_GS,core_) because the tunneling is governed through *V*_GS,core_.
2. The trade-off between change in threshold voltage, and linearity in, conductance is observed during depression operation.
3. We can investigate the learning and inference capabilities of the proposed synaptic device for hardware based neural networks (HNN).
4. A reliable and consistent digit recognition accuracy of 92.28% is achieved by a single layer neural network on the MNIST dataset.

Furthermore, the analysis highlights the feasibility of the proposed synaptic device for inference applications pertinent to neuromorphic computing.

## Figures and Tables

**Table 1:** Device specification for CSDG device as synapse.

Device Parameters	Values
Gate length (*L*_g_)	100 nm–50 nm
Silicon core channel radius (*T*_Si_)	20 nm
Tunneling oxide thickness (*T*_TOX_)	2 nm (SiO_2_)
Nitride layer thickness (*T_N_*_OX_)	4 nm (Si_3_N_4_)
Blocking oxide thickness (*T*_BOX_)	6 nm (SiO_2_)
Oxide thickness (*T*_OX_)	2 nm (SiO_2_)
Core-gate workfunction (*ϕ*_m_,_Core_)	4.6 eV
Shell-gate workfunction (*ϕ*_m_,_Shell_)	4.8 eV
Channel doping (*N*_A_)	10^15^ cm^−3^
Source/drain doping (*N*_D_)	10^20^ cm^−3^

## Data Availability

The data presented in this study are available on request from the corresponding author.
